# A milestone reached?

**DOI:** 10.1080/03009734.2017.1403983

**Published:** 2018-01-17

**Authors:** Arne Andersson

When I last commented on the recently released Impact Factor figures I dwelt upon the problem of predatory journals stealing our manuscripts (1). This tricky issue has not declined. Thus, Jeffrey Beall on 15 January 2017 declared very dramatically that he had ‘unpublished’ his list of predatory publishers and journals, which contained more than 1,200 objects at that time. Many people then thought that he had been physically threatened to do so. Some months later he communicated that his employer had forced him to stop working with that project because of its legal ramifications (2). His personal feelings on the future of scholarly publishing are quite clear. There is a war out there: on the one side predatory publishers supported by some librarians offering pay-to-publish services and on the other the traditional scholarly publishing industry retaining the peer review process to assure a high quality of science.

It is therefore a pleasure this time to bring good news. When Clarivate Analytics, now running the former Thomson Reuters (Web of Science), presented the Impact Factor figures for 2016 we were found to have passed the critical 2.0 level, more exactly 2.39. A value that has for long been regarded as the level for an esteemed scientific journal. Moreover, we now belong to the top quartile of journals in the category ‘Medicine, General and Internal’. Our exact position is 37 of 155. Ahead of us are journals like *New England Journal of Medicine* with an Impact Factor figure of 72.41 and *The Lancet* (47.83). A fascinating journey from the bottom quartile in 2008 (82 of 107) when we started our successful collaboration with Taylor & Francis, who provide us with services for electronic manuscripts.

Our challenge now will be to remain at this level. How should we accomplish this? There are four measures that I would like to announce at this time. First, we are very much aware of the fact that speeding up the editorial processes would be greatly appreciated by a majority of authors/scholars. However, we already have unusually short lead times—a mean of 14 days and a median value of five days before the first decision on the fate of submitted original papers. There are examples of scholarly journals that offer their submitting authors a ‘fast track’ opportunity. Usually that means that a decision to accept/revise/reject is given no later than a week after the submission date. Such journals often handle large volumes of manuscripts and have a staff of scientists checking newly submitted papers immediately upon arrival at the editorial desk. Such an organization costs a great deal of money, and authors accordingly have to pay for this service. Our solution to this problem will be to identify good or even outstanding papers at the editorial level and treat them as ‘internal fast track’ submissions with the aim of presenting a first decision within 10 days. A potential problem could be that the traditional peer review process could be jeopardized. Possibly a reviewer may be less than familiar with the research field in question, and conflicts of interest may arise. Overcoming such obstacles could take weeks or even months, which is the price for retaining the traditional peer review procedures and one that has to be taken into account.

Second, we are planning for a special issue for the coming volume. Uppsala Clinical Research Centre will compile a number of articles dealing with various current projects. Stefan James, Johan Sundström, and Lars Berglund will serve as Guest Editors. Without doubt, our special issues have been of the utmost importance not only for the increase of our Impact Factor but also as impressive presentations of high-quality research carried out at our Medical Faculty.

Third, we have once again decided not to implement APCs (article-processing charges) for articles published in our journal. Quite the contrary, we will not charge authors for their color prints in the printed version. Black and white figures/microphotographs are less informative and dull. A favorable offer from Taylor & Francis was difficult to resist. This will start in issue 1, 2018.

Fourth, to initiate a more direct contact with our current and potential authors we are now committing ourselves to social media. Many other journals have already established such activities, mostly as Twitter accounts. One possibility will be to ask authors of accepted papers to release a Tweet on their brand-new discoveries—a much shortened and popularized abstract—that might be more easily caught by various news channels. Likewise, we will have the chance to highlight news on the maintenance of our journal and the fate of different papers. Have a look at UJMS@Arne UJMS, and you will find examples of our first unsure steps along these new publishing avenues.

In this context I would like to call your attention to something named ‘Altmetrics’. For those of you who visit our website now and then to check citation numbers and numbers of views (=downloads) for separate articles you will most certainly have noticed that there is a third score value denoted Altmetrics. To cite the company, ‘Altmetrics can tell you a lot about how often journal articles and other scholarly outputs are discussed and used around the world’. For most of the articles the scores are 0 or 1. That is, no one in social media or the various news channels has paid attention to the article. There have been, however, a couple of remarkable exceptions during recent years in our journal. The most obvious is the Rudbeck Award review article by Otto Cars and colleagues (3) on antibiotic resistance and the threat to the world’s sustainable development it could pose. It has at present an Altmetrics score of 95! Besides being highlighted in an article in *Washington Post* it has been mentioned several times in policy documents (WHO), Tweets, Facebook contributions, and Mendeley bookmarks. Two other high-scoring articles are those by Hughes and Karlén (4) on the development of new antibiotics and the interesting report of an Uppsala–Umeå collaboration on the finding that lumbar spinal stenosis may be a consequence of senile systemic amyloidosis (5). Interestingly, this latter publication received its score points mainly because of a report in *Upsala Nya Tidning* on the study. Needless to say, Åke Spross was the author of the report. Doubtless, this view on the impact of research activities in terms of attention reached in media other than traditional citation-based metrics will become increasingly important.

All these renewal activities will, we hope, contribute to a favorable future for our journal. Here I would like to express my personal view on this perhaps ‘nerdy’ evaluation of scholarly publishing by means of the Impact Factor tool. Admittedly it has many short-comings (6), but there is no better way of doing it at present. So, my answer to the current title question is ‘yes’ but with another question mark because many people claim that the Impact Factor says nothing about the quality of published articles (7). I tend to agree, but still the most common argument for not submitting their papers to our journal, when I try to stimulate our most talented researchers to do so, is that ‘your Impact Factor value is too low’. Therefore, I have to live with the premise that the denominator should not drop any lower, i.e. not accepting papers that are unlikely to be cited; and further trying to find papers that contain news that will make it a ‘high-citation’ paper, increasing the numerator in the impact factor fraction. Thus, let us struggle on with the mission of keeping our old (8) journal vital and vivid to the satisfaction and joy of our colleagues and members of the society.
